# Cutaneous lupus erythematosus in dogs: a comprehensive review

**DOI:** 10.1186/s12917-018-1446-8

**Published:** 2018-04-18

**Authors:** Thierry Olivry, Keith E. Linder, Frane Banovic

**Affiliations:** 1 Department of Clinical Sciences, College of Veterinary Medicine, North Carolina State University, Raleigh, NC 27606 USA; 2 Comparative Medicine Institute, North Carolina State University, Raleigh, NC USA; 3 Department of Population Health and Pathobiology, College of Veterinary Medicine, North Carolina State University, Raleigh, NC USA; 4 Department of Small Animal Medicine and Surgery, College of Veterinary Medicine, University of Georgia, Athens, GA USA

**Keywords:** Auto-immune skin diseases, Auto-immunity, Canine, Dermatology, Lupus, Skin

## Abstract

Since the first description of discoid lupus erythematosus (LE) in two dogs in 1979, the spectrum of canine cutaneous lupus erythematosus (CLE) variants has expanded markedly.

In this review, we first propose an adaptation of the Gilliam-Sontheimer classification of CLE for dogs. We then review the signalment, clinical signs, laboratory and histopathology and treatment outcome of the currently recognized variants of canine CLE, which are vesicular CLE, exfoliative CLE, mucocutaneous LE and facial or generalized discoid LE. We end with a short description of the rare cutaneous manifestations of systemic LE in dogs.

Canine CLE variants are heterogeneous, some of them mirror their human counterparts while others appear—thus far—unique to the dog. As most CLE subtypes seem to have a good prognosis after diagnosis, veterinarians are encouraged to become familiar with the spectrum of often-characteristic and unique clinical signs that would permit an early diagnosis and the rapid implementation of an effective treatment.

## Background

In 1979, Griffin and colleagues were the first to report a skin disease of dogs that resembled discoid lupus erythematosus (DLE), one of the variants of cutaneous lupus erythematosus (CLE) of humans [1]. Within the ensuing two decades, new information was limited to a large case series of canine DLE [2–4] and a catalog of skin lesions present in dogs with systemic lupus erythematosus (SLE) [5]. It was only around the turn of the millennium that other cutaneous variants of canine LE were characterized, notably type I bullous systemic LE, as well as exfoliative and vesicular CLE [6–8]. Finally, a third wave of descriptions of canine CLE subsets occurred more recently with the publication of case series of mucocutaneous LE and generalized DLE in dogs [9, 10].

In this paper, we first propose a classification of canine CLE variants, which is derived from the princepst modern nosology of the corresponding human diseases. This first section will be followed by a series of monographs reviewing relevant information published to date on the various canine CLE subsets.

### Classification of cutaneous lupus erythematosus

#### Classification in humans

In 1997, Gilliam-Sontheimer proposed a nosology that is the modern foundation of the classification of cutaneous manifestations of LE in humans [11]. This system separates skin lesions associated with LE into two groups. Those that have microscopic skin lesions specific for lupus (i.e. a lymphocyte-rich interface dermatitis with basal keratinocyte apoptosis) are named “*LE-specific skin diseases”* (or CLE sensu stricto) while those that do not share such a histopathologic pattern are grouped under the denomination “*LE-nonspecific skin diseases*” [11, 12].

**Fig. 1:**
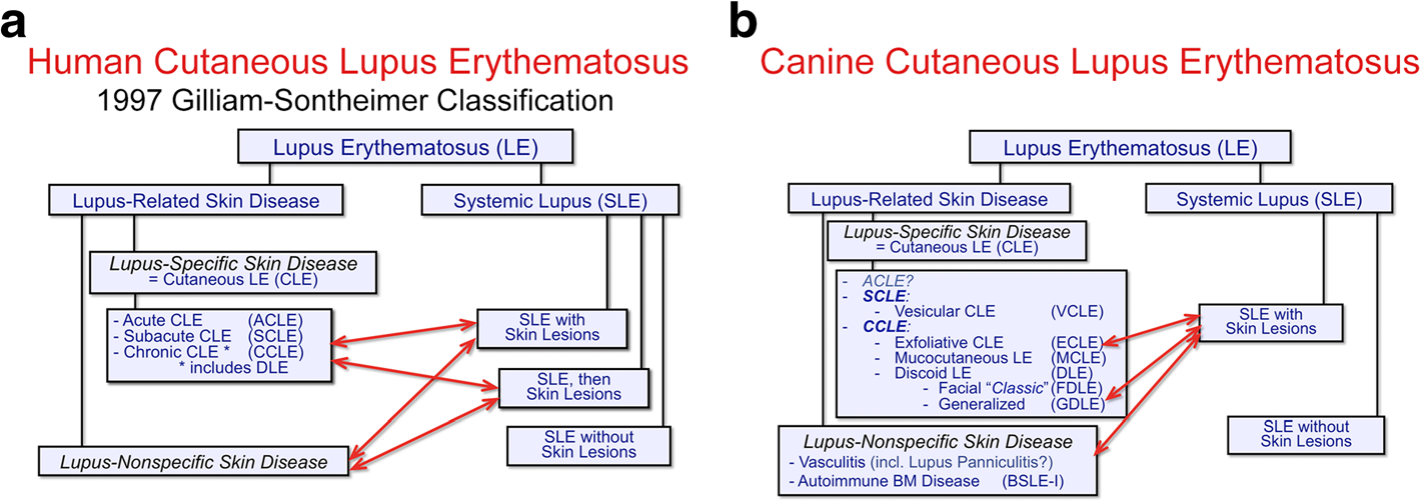
Classification of skin manifestations of lupus erythematosus in humans and dogs. **a** Gilliam-Sontheimer classification of human cutaneous lupus erythematosus variants; **b**: proposed classification of canine cutaneous lupus erythematosus variants

In this classification, LE-specific skin diseases (CLE) are further subdivided into three major subcategories based on the lesional morphology and the average duration of individual skin lesions; these are named acute cutaneous LE (ACLE), subacute cutaneous LE (SCLE) and chronic cutaneous LE (CCLE) (Fig. 1a). Lupus erythematosus-nonspecific skin lesions encompass those associated with the underlying autoimmune disease, but that are not specific for LE itself, since the same lesions can be seen also in other diseases. Examples of LE-nonspecific skin lesions are those due to vasculitis, cryoglobulinemias, or vesicobullous lesions associated with basement-membrane autoantibodies (i.e. bullous SLE).

Importantly, human patients with SLE might exhibit cutaneous lesions that can be either specific or nonspecific (SLE with or without CLE). Conversely, LE-specific skin lesions can be present with or without systemic involvement (CLE with or without SLE) (Fig. 1a).

A simplified version of this classification has been reported recently [13]. A recent review summarizes the salient clinical and diagnostic features of human CLE variants [14].

#### Proposed classification in dogs

It seems logical to use the same logic to classify the cutaneous manifestations of LE in dogs as that first developed by Gilliam and Sontheimer (Fig. 1b). Herein, we also suggest to separate LE-specific skin diseases (CLE *sensu stricto*) from those that are lupus-non-specific. Among CLEs, a canine homologue of ACLE of humans has not yet been reported. In contrast, vesicular cutaneous LE (VCLE) is the only identified canine CLE variant that is an equivalent to human SCLE. Exfoliative cutaneous LE (ECLE), localized (facial) or generalized discoid LE (DLE) and mucocutaneous LE (MCLE) are the currently recognized subtypes of canine CCLE.

At this time, we would also regroup under the umbrella of LE-nonspecific skin diseases the various skin lesions that are seen not only in the context of SLE, but also outside of this syndrome. Examples are vasculitis and the type I-bullous SLE associated with collagen VII autoantibodies (i.e. an epidermolysis bullosa acquisita occurring in the context of SLE); one case of putative “lupus panniculitis” was mentioned  in a case series of cutaneous manifestations of SLE in dogs [5].

## Lupus-specific skin diseases

The salient features of lupus-systemic skin diseases in dogs are summarized in Table 1.

**Table 1:** Comparative characteristics of cutaneous lupus erythematosus variants in dogs

	SCLE	CCLE			
	VCLE	ECLE	MCLE	FDLE	GDLE
Most commonly affected breeds	Shetland sheepdogs, rough collies and border collies	German shorthaired pointers and Magyar viszlas	German shepherd dogs	German shepherd dogs	Chinese crested dogs
Ages of onset: median (range)	5.5 (2.0–11.0)	0.7 (0.2–3.5)	6.0 (3.0–13.0)	7.0 (1.0–12.0)	9.0 (5.0–12.0)
female-to-male ratios	0.9	1.4	1.8	0.7	1.0
Most common skin lesions	figurate erythema, flaccid vesicles and erosions	erythema, scaling, follicular casts, alopecia and occasional scarring	erosions, ulcers with or without peripheral hyperpigmentation	dyspigmentation, erythema, erosions, ulcers, scaling crusting,	dyspigmentation, erythema, erosions, ulcers, scaling, crusting
Most common lesion distribution	abdomen, axillae, medial thighs, concave pinnae and perimucosal areas	trunk, muzzle, pinnae and abdomen	genital, perigenital, anal, perianal, periocular and perilabial areas	nasal planum and dorsal muzzle	trunk, lateral legs and abdomen
Systemic signs	typically not seen	lymphadenomegaly, arthralgia, and reproductive defects	typically not seen	typically not seen	typically not seen
Most relevant clinical mimics	erythema multiforme	sebaceous adenitis	mucocutaneous pyoderma, mucous membrane pemphigoid and erythema multiforme variants	mucocutaneous pyoderma, epitheliotropic cell lymphoma and uveodermatological syndrome	hyperkeratotic erythema multiforme and generalized ischemic dermatopathies

Disease name abbreviations are listed at the end of this paper

## Subacute cutaneous lupus erythematosus

### Vesicular cutaneous lupus erythematosus

#### Historical perspective

First recognized in the late 1960’s, “*hidradenitis suppurativa*” was a unique skin disease described in Collies, Shetland sheepdogs and their crosses [15, 16]. Since the early 1980’s, the disease mentioned above was suspected to represent, in fact, bullous pemphigoid [17, 18] or erythema multiforme in these breeds [19, 20]. In 1995, an "*idiopathic ulcerative dermatosis of Collies and Shetland sheepdogs*" was individualized as a separate entity that was initially linked to juvenile dermatomyositis, also seen in these breeds [21]. In 2001, Jackson and Olivry separated this ulcerative dermatosis from dermatomyositis based on clinical and histological grounds, and the denomination of VCLE was then coined [8]. In 2004, the same authors reported the detection of circulating anti-Ro autoantibodies in dogs with VCLE [22], and they highlighted the similarity of this canine disease with human SCLE.

#### Incidence and prevalence

At this time, there is insufficient information on canine VCLE to appropriately assess the incidence and prevalence of this disease in dogs. However, this entity has been diagnosed in several countries and continents over the last five decades.

#### Signalment

The clinical characteristics of canine VCLE can be inferred from six reports including 25 dogs [23–28]. Among these cases, there were 11 Shetland sheepdogs and their crosses (44%), seven (rough) collies (28%) and seven pure- or cross-bred border collies (28%). The female-to-male ratio was 0.9 and the age of onset varied between 2.0 and 11.0 years of age (median 5.5 years). That VCLE has been recognized almost entirely in collie-related breeds suggests the existence of a strong genetic predisposition, but the genetics of this disease have not yet been elucidated.

#### Clinical signs

**Fig. 2:**
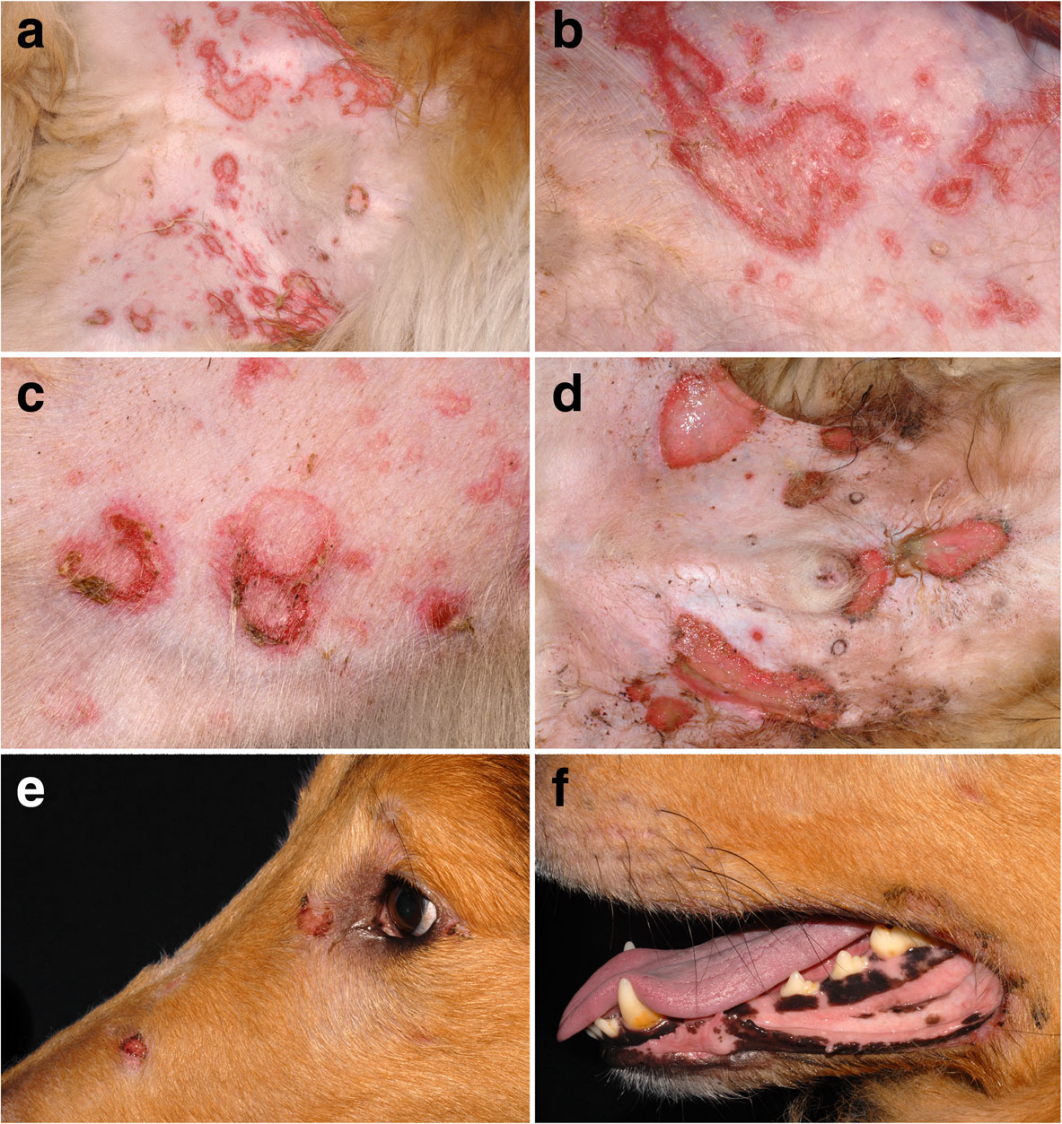
Clinical characteristics of canine vesicular cutaneous lupus erythematosus. **a**, **b**, **c**: erythematous macules progress to annular-to-polycyclic lesions with central flaccid vesiculation and peripheral erythema; skin lesions predominate on the ventral abdomen, medial thighs and axillae. **d**: with chronicity, ulceration can become more prominent. **e**, **f**: erosions at mucocutaneous junctions can be seen in some dogs

Dogs with VCLE present with erythema and flaccid vesicles that slough to leave erosions and ulcers; these predominate on glabrous skin of the abdomen, axillae, groin and medial thighs [8, 23–28]. Skin lesions exhibit a unique sharp-edged annular, polycyclic or serpiginous pattern (Fig. 2a-d). There is accompanying ulceration of mucocutaneous junctions (Fig. 2e,f), concave pinnae and oral cavity in some patients, but these nonventral lesions are usually minor in extent and severity [8, 23–28]. The secondary bacterial colonization of erosive/ulcerative lesions is common. Altogether, these lesions resemble those of the vesicular variant of human SCLE. Pruritus manifestations are usually absent, except, perhaps, for a licking of eroded lesions [23–28].

In eight of 11 (73%) dogs with VCLE, clinical signs were reported to first arise in the summer [23]. In three cases where this information was available, lesions recurred during summer months [23]. Systemic signs are typically not seen in dogs with VCLE, though one dog was reported with weakness and lethargy with associated electromyographic changes interpreted as myositis [24]. There are normally no relevant hematology and clinical biochemistry changes.

The main dermatosis with clinical signs mimicking VCLE is erythema multiforme and its variants.

#### Histopathology

**Fig. 3:**
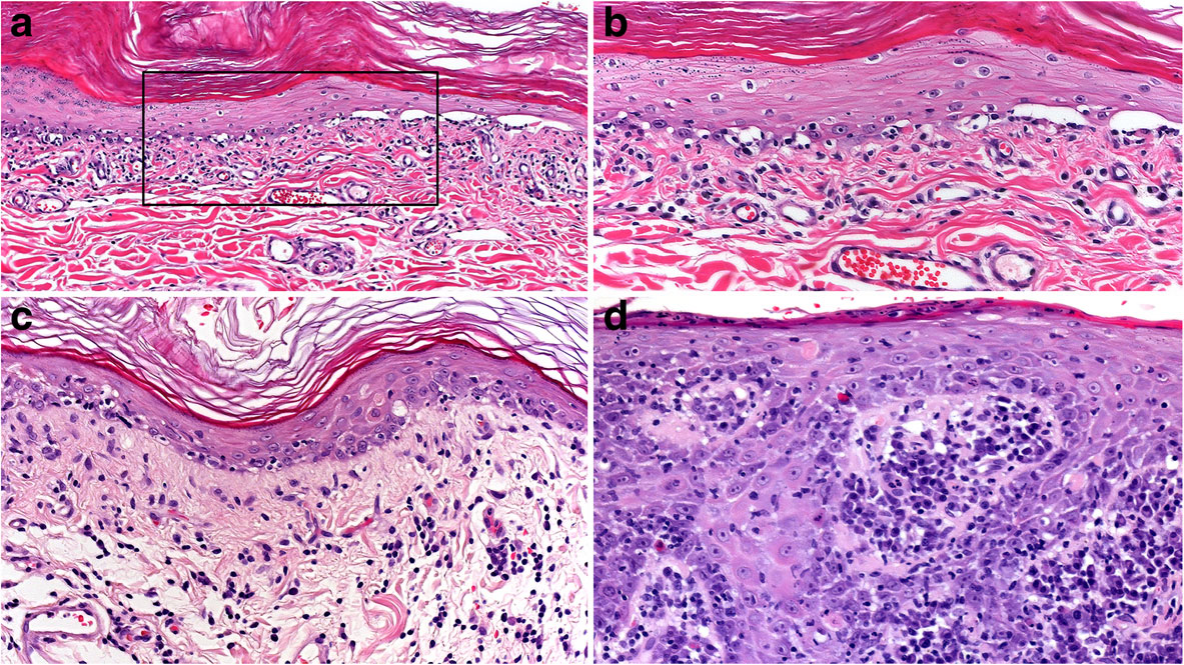
Histopathology of canine vesicular cutaneous lupus erythematosus. **a**: cell-rich, lymphocytic interface dermatitis is present. Marked basal keratinocyte apoptosis has caused a secondary cleft (vesiculation) through the epidermal basal cell layer, which is typical of the disease. 100X (**b**): inset box from image “a”, lymphocytes infiltrate the basal layer and are associated with basal cell vacuolation, apoptosis, loss and disorganization at the cleft margin. 200X (**c**): dermal lymphocytic inflammation can be mild, lacking a clear subepidermal band-like (lichenoid) pattern, but lymphocytes are still observed in the basal epidermal layer in association with basal cell loss. 200X (**d**): chronic lesions can develop epidermal hyperplasia, a prominent dermal infiltrate of lymphocytes and plasma cells and thickening of the basement membrane zone. 200X

In canine VCLE, a lymphocyte cell-rich interface dermatitis is associated with prominent basal keratinocyte vacuolation, apoptosis and loss, which is often sufficient to cause intrabasal clefts and epidermal vesiculation, typical of the disease (Fig. 3a-c) [8]. Basal cell apoptosis is reported to be as high as 16 apoptotic basal cells per 1 mm of epidermis utilizing immunohistochemical detection methods [23]. Hair follicle infundibula have a similar lymphocytic interface and mural folliculitis [8]. Pigment dispersal to dermal macrophages (pigmentary incontinence) is often not a feature or is very mild, likely due to breed coat coloration and the tendency for lesions to occur in poorly- or non-pigmented skin. The thickening of the basement membrane zone and superficial dermal fibrosis are uncommon, which is attributable to the subacute nature of the disease, but they can occur in persistent lesions (Fig. 3d). Cell-rich lesions dominate biopsies but very mild lymphocytic dermal infiltrates or even cell-poor areas of lesions can occur that lack a subepidermal, band-like (lichenoid), dermal infiltrate of lymphocytes (Fig. 3c) [8]. Cell-poor areas of lesions can lead to confusion with juvenile dermatomyositis, which is seen often in the same breeds [8]. Dermatomyositis presents with lesions of ischemic dermatopathy (i.e. cell-poor interface dermatitis and ischemic follicular atrophy), but cell-poor VCLE lesions have more lymphocyte exocytosis into the basal epidermal layer, with lymphocytic satellitosis of apoptotic basal keratinocytes. If the intrabasal level of epidermal clefts is not recognized (Fig. 3b), then vesiculation can be confused with subepidermal autoimmune blistering skin diseases such as mucous membrane pemphigoid (MMP), bullous pemphigoid (BP) and epidermolysis bullosa acquisita (EBA). The prominence of basal apoptosis and intrabasal epidermal vesiculation, when present, supports the histological diagnosis of VCLE over that of other variants of CCLE, but this distinction is difficult for more chronic lesions and is best done clinically, as for all forms of canine CLE. Occasionally superficial epidermal apoptosis with lymphocytic satellitosis might erroneously suggest the diagnosis of erythema multiforme and its morphologically related conditions [29]. Neutrophilic inflammation is common in lesions that progress to ulcers and support the development of secondary bacterial infection.

#### Immunohistochemistry

In one of the two largest case series [22], detailed information on mononuclear cell immunophenotyping was reported. T-lymphocytes expressing CD3 were found in epidermal sections of all 11 dogs examined. In two of these dogs with VCLE, the phenotype of skin-infiltrating leukocytes was similar: approximately 25 to 50% of epidermal leukocytes were T-lymphocytes expressing the alpha-beta T-cell receptor, CD3 and CD8; less commonly, epitheliotropic lymphocytes expressed CD4. The other epithelial leukocytes were identified as CD1-positive Langerhans cells. In the superficial dermis, infiltrating cells consisted of an approximately equal population of alpha-beta T-lymphocytes expressing CD4 or CD8-alpha and CD1-positive dermal dendritic cells. Rare CD21-positive B-lymphocytes were detected in the superficial dermis. In contrast, gamma-delta T-cells were not identified in either the epidermis or dermis. Basal keratinocytes expressed high levels of ICAM-1 and low levels of class II major histocompatibility complex molecules signifying their activated state. In this study, apoptotic keratinocytes were observed in the basal epidermis of seven of the 12 dogs evaluated (58%) [22].

#### Immunopathology

##### Direct immunofluorescence

Direct immunofluorescence revealed the presence of IgG at the basement membrane zone in 7/14 (50%) dogs with VCLE [22]. The deposition of IgG around blood vessels was observed in 13/14 dogs (93%). Finally, cytoplasmic basal keratinocyte IgG was detected in 6/14 subjects (43%); the deposition of activated complement was not seen [22].

##### Indirect immunofluorescence

Indirect immunofluorescence did not reveal anti-basement membrane circulating IgG autoantibodies in the serum of five dogs with VCLE [22]. Similarly circulating antinuclear IgG autoantibodies were not detected in the serum of any of 11 dogs with VCLE using human Hep2 cells as a substrate [22].

##### Immunoblotting and ELISA

Using Hep2 cell extracts, immunoblotting permitted the detection of autoantibodies against soluble nuclear antigens in 9/11 tested sera (82%) [22]. When an ELISA was performed with purified human soluble nuclear antigens, the serum from 8/11 dogs with VCLE (73%) was found to have IgG autoantibodies that bound to these antigens. Antibodies were found to target Ro/SSA (45% of dogs), La/SSB (45%), Sm/RNP (45%), Scl70 (36%), Jo-1 (36%) and Sm-SnRNP (18%) [22]. Overall, and as seen in humans with SCLE, most dogs with VCLE (6/11; 55%) were found to have IgG antibodies that targeted Ro/SSA and/or La/SSB antigens [22].

#### Treatment and outcome

As VCLE is induced and/or worsened by UV light, sun avoidance should be implemented immediately after a diagnosis is made. The first case series provided detailed information on the post-treatment outcome in 11 dogs with VCLE [23]. In six of these dogs (55%), clinical signs resolved with the oral administration of prednisone at low immunosuppressive dosages (2 mg/kg/day), which were tapered according to treatment response. In three dogs (27%), azathioprine (at about 2 mg/kg/day) was added to the treatment regimen due to the insufficient reduction of lesions with glucocorticoids. Finally, the response to pentoxifylline (initially prescribed due to the then erroneous inclusion of VCLE in the dermatomyositis spectrum) was reported as poor in four dogs (36%). In this case study of 11 dogs, one (9%) died of unknown cause, and three (27%) were euthanized at the owner’s request due to poor response to treatment. In the remaining seven dogs (64%), a complete or sub-complete remission of signs was achieved with glucocorticoids alone or in combination with azathioprine [23]. Lesions have also been shown to respond to the immunosuppressant mycophenolate mofetil in one rough collie with VCLE, as the introduction of this drug led to the complete remission of skin lesions after the discontinuation of oral glucocorticoids [27].

More recently, the benefit of calcineurin inhibitors, which had been previously reported in two dogs with VCLE [24, 26], was confirmed in 11 additional patients [28]. In all dogs, treatment was initiated with sun avoidance, oral glucocorticoids and oral ciclosporin at a median dosage of 5.5 mg/kg/day. A complete remission of skin lesions occurred in 8/11 dogs (73%) within one to two months of starting treatment. In two dogs (18%), lesion remission was achieved by increasing the dose of ciclosporin and adding topical 0.1% tacrolimus ointment. While relapses of clinical signs were common when the dosage of ciclosporin was lowered, the long-term remission of signs was possible with calcineurin inhibitors, either alone or in combination. These observations suggest that calcineurin inhibitors might be the drug category of choice to treat canine VCLE.

## Chronic cutaneous lupus erythematosus

### Exfoliative cutaneous lupus erythematosus

#### Historical perspective

In 1992, Ihrke, Gross and Walder described a scaly dermatosis in young German shorthaired pointers (GSHP). Because microscopic lesions resembled those seen in subjects with lupus, the disease was named “*hereditary lupoid dermatosis*” [30]. One brief case report [31], one series of five cases [32] and a book chapter [33] constituted the early descriptions of this rare disease.

In 1999, we reviewed the histopathological and immunological characteristics of eight dogs with this disease, and proposed the name exfoliative cutaneous lupus erythematosus (ECLE) [7]. Clinical, histopathological and immunological data from 25 dogs with ECLE were later collated and described in more detail [34].

#### Incidence and prevalence

At this time, there is insufficient information on canine ECLE to appropriately assess the incidence and prevalence of this disease in dogs. It appears to have a worldwide distribution.

#### Signalment

This variant of CCLE is predominantly seen in GSHPs [34]. A large pedigree analysis of 235 purebred GSHPs and experimental mating studies established that this disease was transmitted on an autosomal recessive manner [35]. A single nucleotide polymorphism on the CFA 18 chromosome was found to perfectly segregate with the trait in 267 dogs [35]. Interestingly, ECLE has been diagnosed also in several Magyar viszlas living in western Europe [36, 37]; this observation is noteworthy, as viszlas share a common ancestry with GSHPs [37].

Adding the cases from the largest case series [34] to those of the genome-wide association study [35] yielded 45 GSHPs already reported with ECLE: there were 26 females and 19 males with a female-to-male ratio of 1.4. The first clinical signs usually occurred in juveniles or young adult dogs with a median age of onset of 8 months (range: 7 weeks to 3.5 years) [32, 34].

#### Clinical signs

**Fig. 4:**
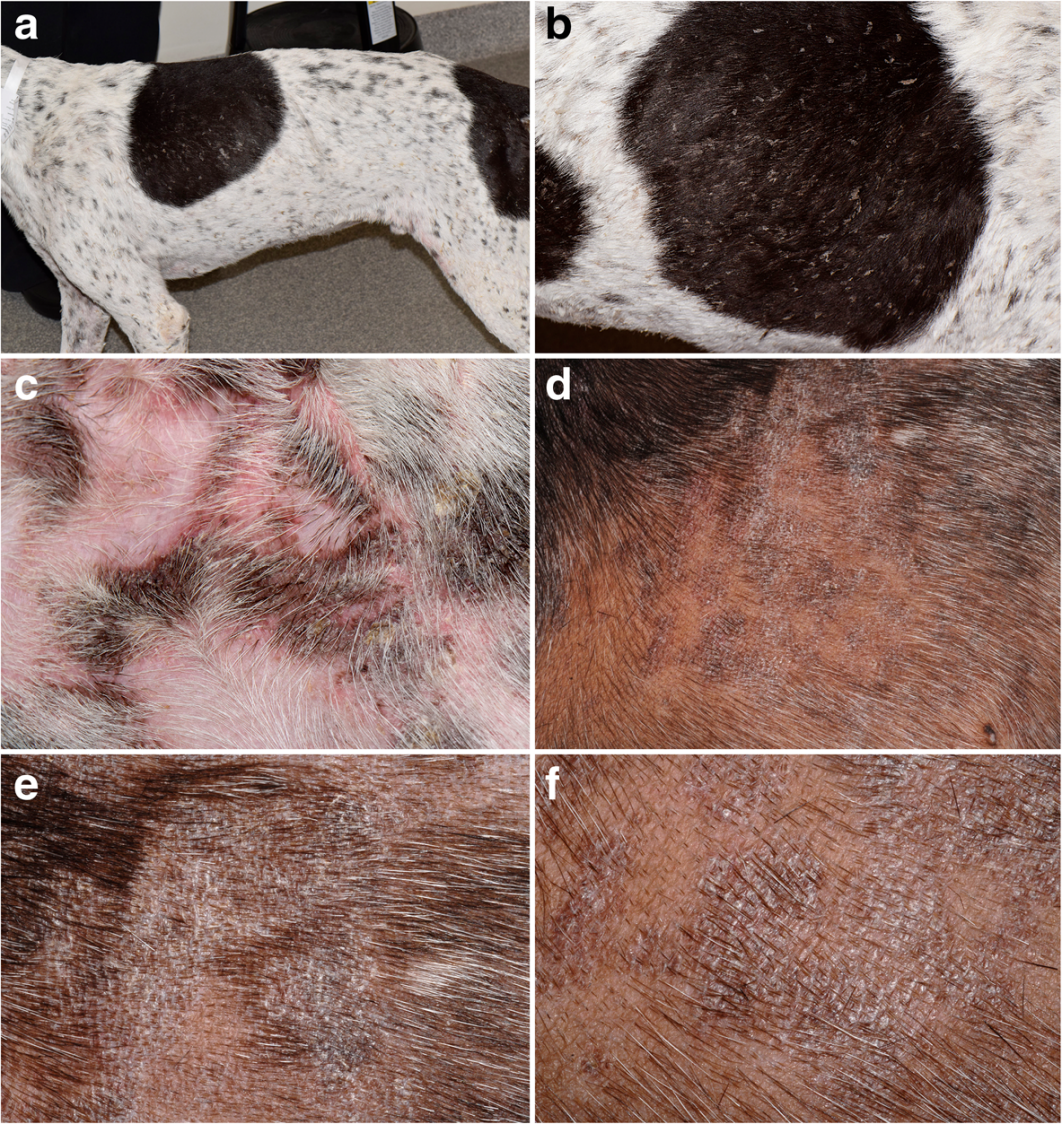
Clinical characteristics of canine exfoliative cutaneous lupus erythematosus in German shorthaired pointers. **a**, **b**: poor hair coat, scaling and follicular casts are visible from a distance. **c**, **d**, **e**, **f**: irregular plaques with hyperpigmentation and scaling can be seen on closer examination - (**d**-**f**) courtesy of Petra Bizikova, NC State University

In the largest clinical case series of ECLE in GSHPs [34], the most prominent skin lesions were scaling and alopecia, which affected 25 (100%) and 19 (76%) of the reported dogs, respectively (Fig. 4a,b). Follicular casts were noted in one third of patients (Fig. 4a,b). Recently seen GSHPs with ECLE were found to also exhibit irregular and polycyclic patches and plaques with dyspigmentation and some scarring (personal observations; Fig. 4c,f). In this form of canine CCLE, skin lesions typically affect the muzzle, pinnae and dorsal trunk and then progress to involve the limbs, sternum and ventral abdomen. Generalized skin lesions are found in most dogs, while crusting, with or without an underlying ulceration, was recorded in one fourth of patients in the largest series of GSHPs [34]. In one dog of that report, ulcers were so extensive that they resulted in bacterial septicemia. Mild pruritus was recorded in one third of GSHPs with ECLE [34].

**Fig. 5:**
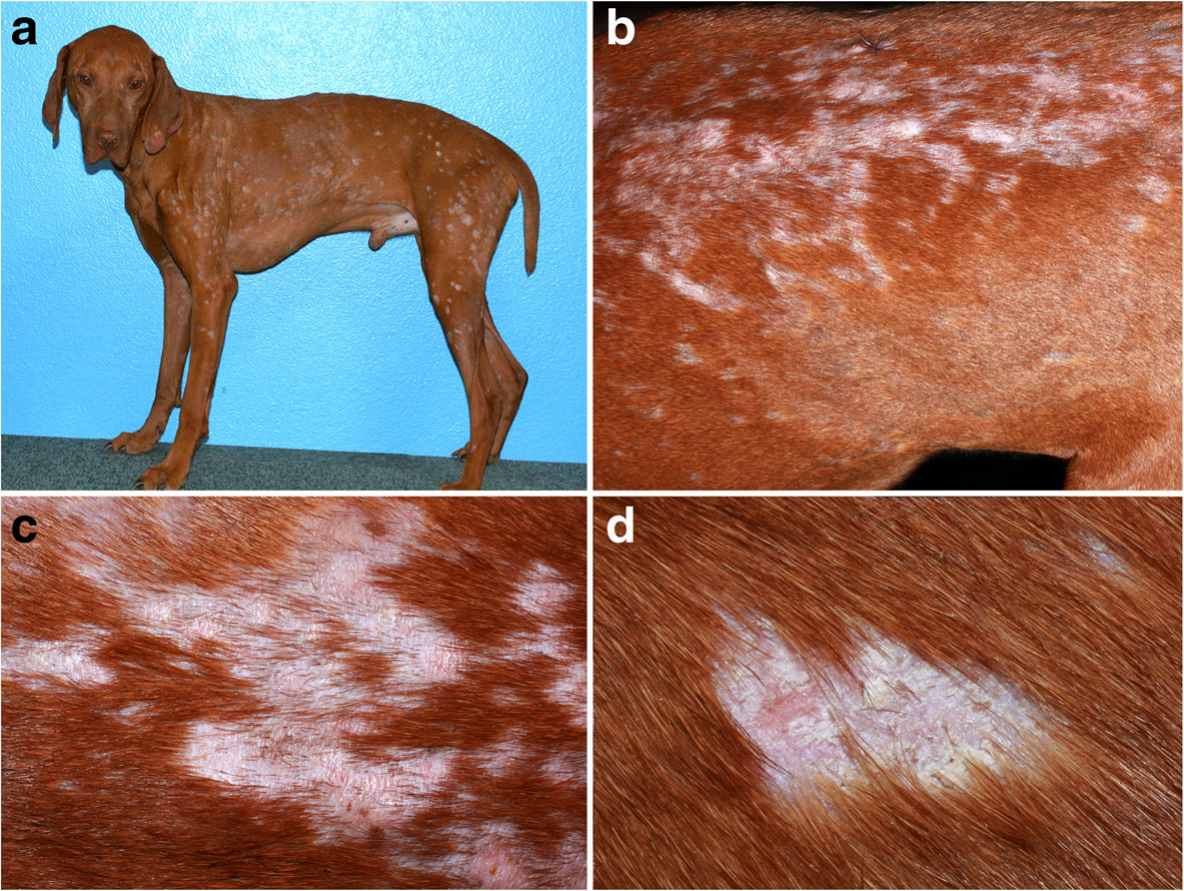
Clinical characteristics of canine exfoliative cutaneous lupus erythematosus in Magyar viszlas. **a**, **b**: multifocal, often coalescing, patches of alopecia are noted from afar. **c**, **d**: atrophic scars and follicular casts and large scales develop in alopecic areas - courtesy of Émilie Vidémont, University of Lyon, France

Overall, skin lesions of ECLE in viszla dogs are nearly identical to those seen in GSHPs with the same disease (Fig. 5a-d). Furthermore, in some viszlas, the alopecic lesions are circumscribed and resemble those of the so-called “*sebaceous adenitis of viszlas*” (Fig, 5a,d). This observation, as well as the presence of typical histological changes of CLE in these dogs, raises the suspicion that some of the viszlas reported with sebaceous adenitis might have had, in fact, ECLE. In fact, in both GSHPs and Magyar viszlas, (granulomatous) sebaceous adenitis is the perfect mimic for ECLE.

A generalized peripheral lymphadenomegaly was reported in one-third of GSHPs with ECLE [34]; lymph node enlargement was also described in other reports [31, 32, 38]. Many GSHPs with ECLE eventually develop signs suggestive of arthralgia, which manifests as a stiff gait, lameness or an arched back [34, 38, 39] In one report, all six dogs were infertile, with azoospermia and irregular or arrested cycles in females [38].

#### Laboratory evaluation

While rare GSHPs with ECLE have mild anemia, fluctuating thrombocytopenia is seen more commonly in these dogs [34, 38]; serum biochemistry and urinalysis usually do not exhibit consistent changes, except for hyperglobulinemia seen occasionally [34, 38].

Fine needle aspirate material from enlarged peripheral lymph nodes was submitted for cytological evaluation in one GSHP with lymphadenomegaly, and it revealed lymphoid hyperplasia. Spinal radiographs, myelogram and cerebrospinal fluid analysis and stifle and hock joint aspirates were performed in dogs suffering from intermittent arthralgia, but they failed to identify any underlying abnormality [34].

#### Histopathology

**Fig. 6:**
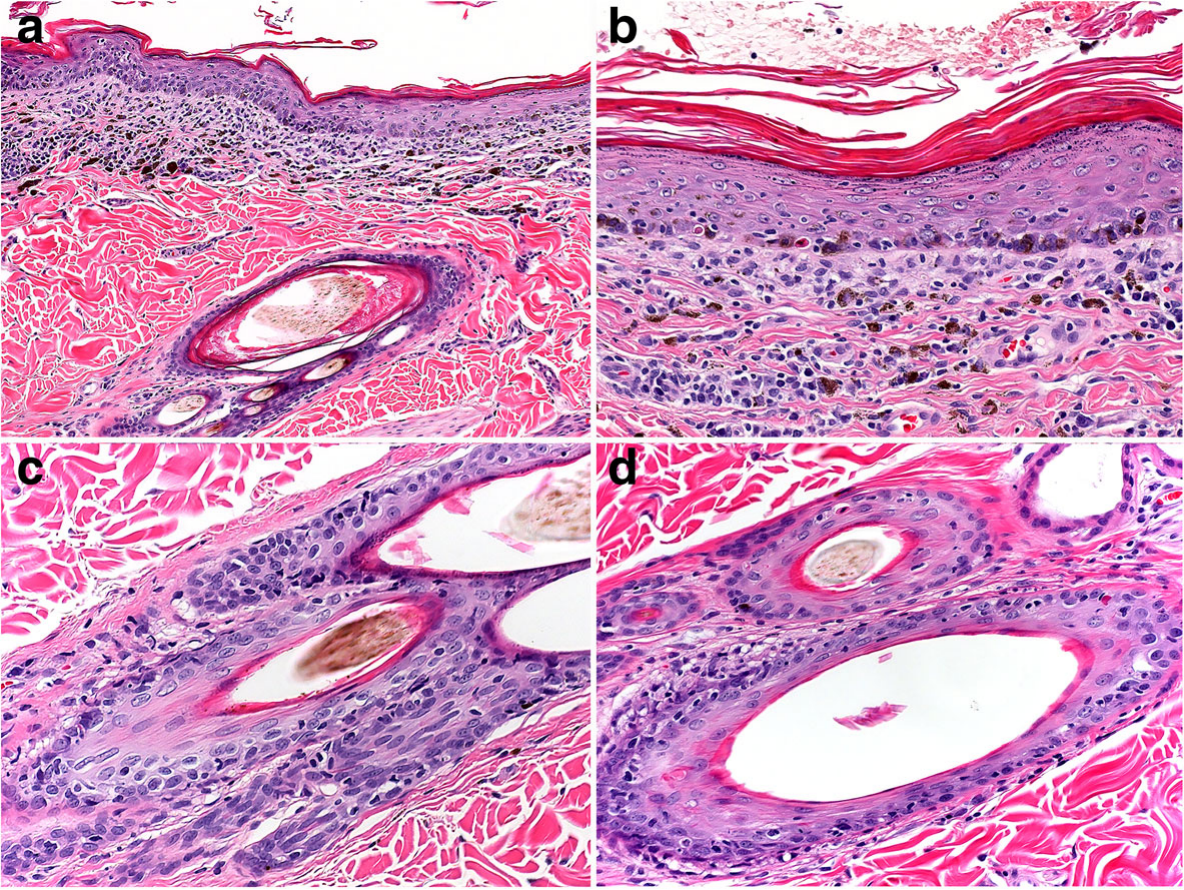
Histopathology of canine exfoliative cutaneous lupus erythematosus. **a**: cell-rich, lymphocytic interface dermatitis is present with a distinct band-like (lichenoid) dermal infiltrate of lymphocytes, plasma cells and a few histiocytes. 100X (**b**): in an area of well-developed interface dermatitis, laminated, orthokeratotic hyperkeratosis (exfoliation) is present, which is typical of the disease. 200X. **c**: lymphocytic interface folliculitis and mural folliculitis involve the infundibulum (upper-right) as well as the isthmus and inferior segments (lower-left) of hair follicles. Sebaceous glands are absent in this biopsy, as is reported in some cases. 200X (**d**): lymphocytic interface folliculitis and mural folliculitis are present in the external root sheath of anagen hair follicles. Telogen hair follicles can also be affected (not shown). 200X

The largest compilation of dogs with ECLE confirms previous information regarding the histopathology of this disease [34]. In this study, microscopic examination revealed a cell-rich interface dermatitis (Fig. 6a,b) characterized by moderate to marked dermal lymphocyte infiltrate that tended to be multifocal, rather than always organized into a subepidermal band. Typical of cell-rich interface lesions, the apoptosis of basal keratinocytes was accompanied by moderate to marked lymphocytic exocytosis in the lower epidermis (Fig. 6b). In addition, biopsies of most dogs had mild lymphocytic exocytosis and keratinocyte apoptosis in the upper epidermis. Diffuse orthokeratotic hyperkeratosis was a notable feature of most biopsies and was usually moderate (Fig. 6b).

In the study by Bryden and colleagues, a lymphocytic interface mural folliculitis was also present in the infundibulum in all dogs, for which biopsy sections captured the infundibula of follicles, and it extended to inferior follicular segments in 92% of dogs [34] (Fig. 6c,d). Sebaceous glands were also affected. A periglandular lymphocytic infiltrate was present in 63% of dogs, sebaceous glands were absent in 50% of all biopsy sections evaluated, and 16% of dogs lacked sebaceous glands in all biopsies (Fig. 6c) [34]. These latter features can lead to confusion with (primary) sebaceous adenitis. Additionally, a lymphocytic apocrine gland infiltrate was observed in 46% of dogs [34].

#### Immunopathology

##### Direct immunofluorescence

In one study [34], direct immunofluorescence testing performed on paraffin-embedded sections revealed the presence of in situ deposition of IgG, IgM, IgA and C3 in the epidermal basement membrane of 100%, 47%, 11% and 5% of GSHPs, respectively. The multifocal or continuous fine deposition of IgG was recorded in 61%, 35% and 77% of skin biopsy sections, respectively. Interestingly, the follicular basement membrane deposition of IgG was found in 41% of tested biopsies.

##### Indirect immunofluorescence

Indirect immunofluorescence testing on sections of normal canine haired and salt-split-skin revealed the existence of circulating anti-follicular IgG antibodies in the serum of 57% of tested GSHPs with ECLE [34]. In addition, anti-sebaceous gland IgG antibodies were also detected in these dogs. Circulating anti-epidermal basement membrane antibodies were not observed, however. In three studies, antinuclear antibody serology usually remained below positive thresholds in GSHPs with ECLE [32, 34, 38].

##### Immunohistochemistry

Immunohistochemical staining confirmed the predominance of CD3-bearing T lymphocytes in the lower epidermis, superficial dermis, in the infundibulum of hair follicles and around sweat glands [34]. These CD3-positive T lymphocytes infiltrated sebaceous glands and their associated ducts in samples collected from two dogs.

##### Treatment and outcome

The review of published reports has yielded inconsistent information on the treatment and outcome of this disease. The early descriptions of ECLE suggested some benefit of dietary changes, supplementation with fatty acids, anti-seborrheic shampoos, antibiotics and/or oral retinoids [31, 32] The most recent case series [34, 38] reported the limited efficacy of immune-modulating drugs prescribed either as single or combination therapy (e.g. tetracycline-niacinamide combinations, doxycycline, oral glucocorticoids, azathioprine, ciclosporin, leflunomide, or hydroxychloroquine).

Hydroxychloroquine, an first-line antimalarial drug used in human CCLE, appeared to slow down the clinical progression in some dogs with ECLE; in contrast, high-dose ciclosporin reportedly was not able to halt lesion worsening [38]. As the response to immunomodulators is heterogeneous in human CCLE variants [40], the use of high-dose oral glucocorticoids and adjunctive immunosuppressive regimens need to be investigated on an individual patient basis [34, 38, 39].

Taking into account all GSHPs with ECLE for which a long-term outcome has been reported [31, 32, 34, 38, 39], over half of dogs are eventually euthanized for their lack of disease response to therapy. This makes this CLE variant the most challenging to treat among all those of canine CCLE.

#### Mucocutaneous lupus erythematosus

##### Historical perspective

In the mid 1990’s, two German shepherd dogs (one in France and one in Québec, Canada) were described as having a genital-predominant DLE [41, 42]. In 1998, we proposed the disease name of MCLE for dogs with perimucosal ulcerative lesions and microscopic characteristics of CLE (Olivry T: British Veterinary Dermatology Study Group, York, 1998). Additional cases with identical phenotypes were later published with the diagnoses of MCLE [43], DLE [44], or, more recently, perianal/perivulvar LE [45]. Finally, we reported a large series of 21 additional dogs with MCLE was reported in 2015 [9] and a single case report from Chile was later published in 2017 [46].

##### Incidence and prevalence

There are no available data to estimate the incidence of prevalence of MCLE in dogs.

##### Signalment

Collating the signalment of all published cases of canine MCLE yielded pertinent information. Of the 36 dogs [9, 41–46], there were 17 German shepherd dogs and their crosses (47%); adding the two Belgian shepherds [43] leads to about half of the dogs with MCLE belonging to breeds related to German shepherds. Altogether, females appear nearly twice over-represented with a female-to-male ratio of 1.8; there was an equal representation of intact and neutered individuals. Interestingly, this female-to-male ratio increases to 3.8 if we only collate data from German/Belgian shepherds and their crosses. In all, the age of onset of skin lesions of MCLE varied between 3 to 13 years (median and means: 6 years). Most dogs for which this information was available (17/28; 61%) began exhibiting noticeable mucocutaneous lesions in mid-adulthood (i.e. between 4 and 8 years of age).

Odds ratios for breed, sex or age predispositions for the development of MCLE cannot be estimated, as dogs come from multiple continents (North and South America, Japan, Europe), and a reference population therefore is not available.

##### Clinical signs

The owners of dogs with MCLE often report perimucosal ulcerative skin lesions with vocalization suggesting pain why defecating or urinating.

**Fig. 7:**
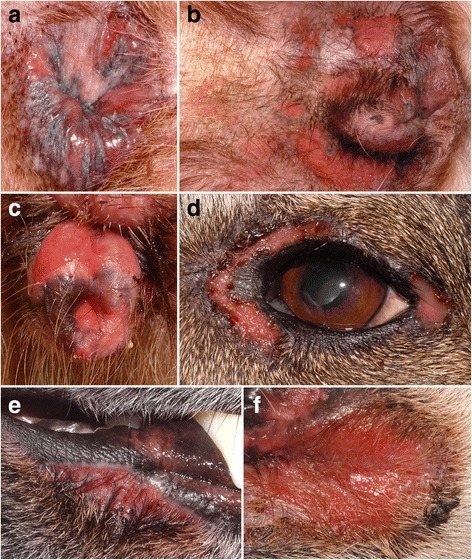
Clinical characteristics of canine mucocutaneous lupus erythematosus. **a**: anal erosions with peripheral hyperpigmentation in a German shepherd dog; (**b**): multifocal perigenital erosions with peripheral hyperpigmentation are often seen in female German shepherd bitches; (**c**): erosions on the lateral sides of the vulva in a German shepherd bitch (courtesy of Pablo Del Mestre, Mar Del Plata Argentina); (**d**): periocular erosions in a German shepherd – these lesions were bilateral (courtesy of Petra Bizikova, NC State University, Raleigh; (**e**): erosion abutting the lip in the same German shepherd dog as in (**a**); (**f**): same dog as in (**b**) – large perilabial erosion; this lesion was also symmetric

At the time of presentation to the veterinarian, lesions have been reported to occur most commonly on or around the anus (24/36; 67%) (Fig. 7a) or on the genitalia or perigenital region (17/36, 47%) (Fig. 7b,c) [9, 41–46]. Similar lesions can also be seen, but less commonly, abutting the lips, but they usually do not cross into the mucosa itself (10 dogs; 28%) (Fig. 7e,f). More rarely, lesions have been noted around the eyes (6 dogs; 17%) (Fig. 7d) and nasal planum (4 dogs; 11%); oral lesion are rarest (3 dogs; 9%) [9, 41–46]. In the largest case series, most dogs had two or more areas affected, and the lesions were usually symmetrically distributed [9].

The characteristic lesions of MCLE are erosions and ulcers (Fig. 7a-f), but the latter do not tend to heal with scarring [9, 41–46], an important difference with the lesions of facial and generalized DLE. Crusts are present when lesions extend into haired skin. Hyperpigmentation can be seen often around ulcerative lesions or at the site of previous ones, thus leaving a figurate or reticulated pattern [9, 41–46]. Pruritus is normally absent or mild, but pain is described when defecating and urinating or when touching the lesions; systemic signs have not been reported [9, 41–46].

The most relevant clinical differential diagnoses of MCLE are mucocutaneous pyoderma (MCP), MMP and EM variants.

##### Histopathology

**Fig. 8:**
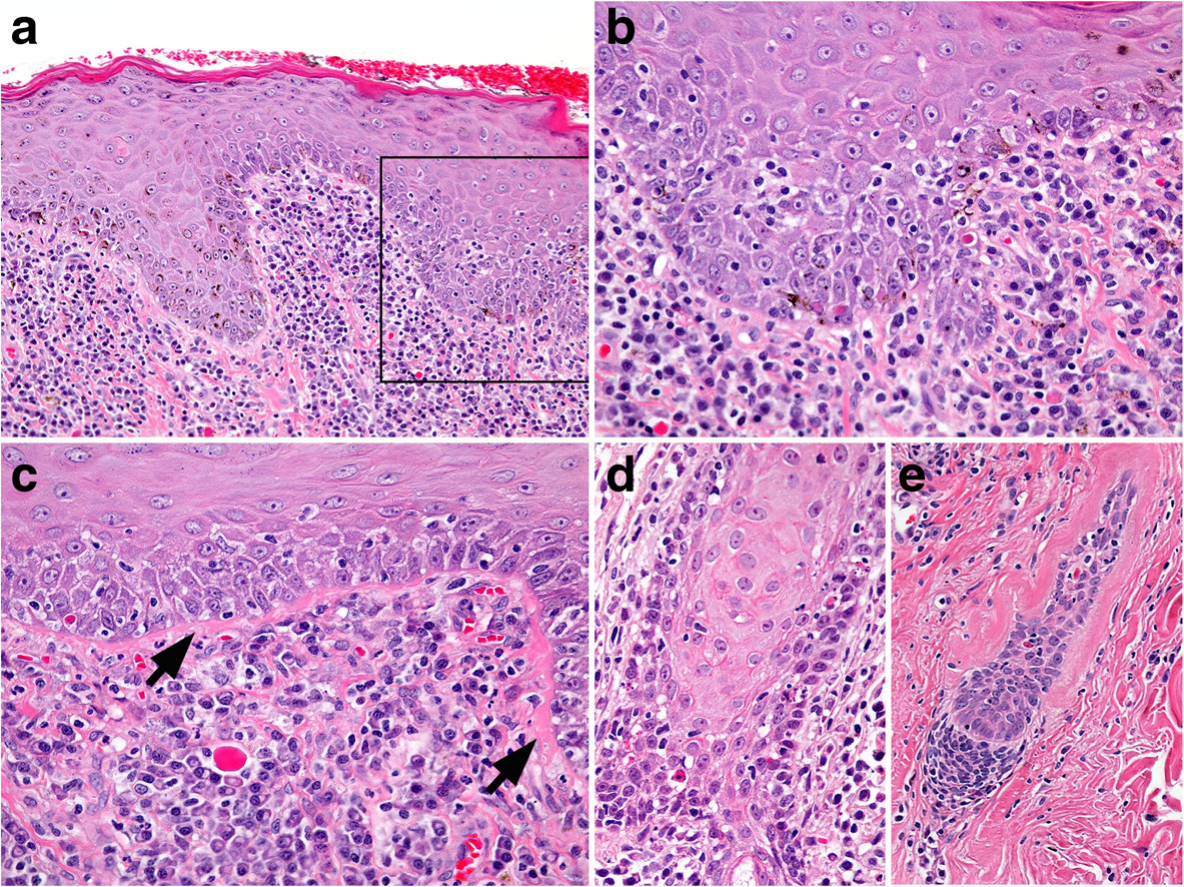
Histopathology of canine mucocutaneous lupus erythematosus. **a**: cell-rich, lymphocytic interface dermatitis is present with numerous plasma cells, including Mott cells, which is common with inflammation in perimucosal skin and is exacerbated by secondary bacterial infection. 100X (**b**): inset box from image “a”, lymphocytes infiltrate the basal and suprabasal layers of the epidermis in association with multifocal basal cell apoptosis. 400X (**c**): basement membrane thickening (arrows) is present and is usually patchy and multifocal. 400X (**d**): lymphocytic interface folliculitis and mural folliculitis involve the infundibulum and extend to the isthmus (not shown) of a hair follicle. 400X (**e**): lymphocytic mural folliculitis of the inferior hair follicle (external root sheath), with apoptosis and follicular atrophy. 200X

In the largest case series, and per inclusion criteria, skin biopsies contained a cell-rich lymphocytic interface dermatitis with basal keratinocyte damage (i.e. basal cell apoptosis, loss and/or hydropic degeneration) [9] (Fig. 8a-c). This pattern was often patchy, or in limited areas, sometimes only being observed at close proximity to an ulcer margin. Interface dermatitis commonly extended to the infundibula of hair follicles (Fig. 8d), while inferior segments of hair follicles are sometimes also involved (Fig. 8e). Basement membrane thickening was found to be multifocal, patchy to diffuse (Fig. 8c). Pigmentary incontinence varied from mild to marked. Plasma cells were present in all cases (Fig. 8b,c), mixed with lymphocytes and were often numerous in subepidermal, perivascular, periadnexal and in dermal areas below erosions and ulcers. Erosions and ulcers were common but granulation tissue was limited and fibrosis (scarring) was not seen. Occasional suprabasal keratinocyte apoptosis was noted in half of the cases, but suprabasal lymphocytic satellitosis, when present, was always mild. Nonetheless, superficial keratinocyte cell death can lead to confusion with EM  and morphologically related conditions. Not surprisingly, for a perimucosal ulcerative disease, lesions of concurrent bacterial infection were common, including neutrophilic crusting, pustules, perifolliculitis and folliculitis, as well as presence of bacteria in surface exudates. Such infection will complicate the histological diagnosis and the successful treatment of pyoderma is warranted prior to biopsy.

##### Immunopathology

In dogs in whom this information was reported, direct IF almost always revealed a positive IgG lupus band test (LBT) [9, 44]. Positive LBTs were sometimes also uncovered for IgA, IgM and C3. Positive ANA titers were rarely found, however.

##### Treatment and outcome

The skin lesions of canine MCLE appear to respond best to immunosuppressive dosages of oral glucocorticoids [9, 41–46]. The complete remission of signs is generally obtained within one month of treatment induction [9]. A combination of a tetracycline antibiotic, with or without niacinamide, appears beneficial either alone or as adjunctive combination in some dogs [9, 41, 45]. In most patients, the tapering of oral glucocorticoids leads to the prompt relapse of skin lesions, which will undergo remission once the dosage is re-escalated again. The usefulness of adding additional immunosuppressive drugs (e.g. azathioprine, ciclosporin, mycophenolate mofetil etc.) to permit the reduction of oral glucocorticoid doses needs further investigations.

#### Discoid lupus erythematosus

##### Historical perspective

Among the several variants of human chronic CLE (e.g. discoid LE [DLE], verrucous (hyperkeratotic) LE, chilblain LE, lupus tumidus and lupus profundus), DLE represents the most common form: it is divided into a localized variant where skin lesions are confined to the head and neck, and a generalized form, in which skin lesions also occur below the neck [47].

In 1979, Griffin and colleagues reported clinical, histopathological and immunological characteristics of two dogs with localized facial lesions that were diagnosed as being affected with the canine counterpart of human DLE [1]. In these two dogs, the nasal-predominant dermatitis was associated with microscopic focal interface dermatitis, basement membrane thickening and a superficial lymphocytic and plasmacytic dermatitis. Since then, there were three large case series describing dogs with nasal skin-predominant DLE lesions [2–4], two of them including some of the same cases [2, 4]. The then-proposed terminology resulted in the widespread acceptance of “*canine DLE*” being equated mainly to facial localized lesions. In the 2010s, we began reporting dogs with a more widespread phenotype that resembled that of the generalized variant of human DLE [48–50]; this was followed with the publication of a case series of ten dogs with generalized DLE (GDLE) [10], this article encompassing the three cases already published by the NC State Dermatology group [48–50].

##### Signalment

The four largest series of dogs with the “classic” localized facial-predominant DLE (FDLE) allows the analysis of a cohort of 104 dogs [3, 4, 45, 51]. Among these cases, there were 32 German shepherd dogs and their crosses (31%). The age of onset of FDLE skin lesions varied between 1 and 12 years of age (median: 7 years); while the female-to-male ratio was 0.7, there was an equal representation of intact and neutered individuals.

A retrospective study recently evaluated the historical and outcome information in ten dogs with GDLE [10]. Amongst these dogs, there were two Chinese crested dogs and two Labrador retrievers; there was one each of the following pure breeds: miniature pinscher, Leonberger, Shih-Tzu and toy poodle. The age of onset of GDLE skin lesions varied between 5 and 12 years of age (median 9 years). The female-to-male ratio was 1.0 and all dogs were castrated. Interestingly—and surprisingly—German shepherd dogs, a breed predisposed to develop several forms of LE, such as SLE, localized FDLE and MCLE, did not seem affected by GDLE. This discrepancy may be explained by the German shepherd breed not being predisposed to this disease, by the small size of the reported cohort or by a possible clinical misdiagnosis of GDLE as one of the” *idiopathic lichenoid dermatoses*” as they were diagnosed in the 1980’s solely based on the histopathological identification of a “lichenoid tissue reaction” in dogs [52].

##### Incidence and prevalence

At this time, there is no usable information to determine the frequency of occurrence of FDLE and GDLE in dogs.

##### Clinical signs

The classic skin lesions of human DLE usually consist of early erythematous and variably scaly macules or papules that slowly evolve into a coin-shaped (i.e. discoid), plaques with adherent scales, follicular plugging (i.e. comedones) and peripheral hyperpigmentation presumed to occur secondarily to inflammation [47]. These discoid plaques can coalesce and develop central scarring and depigmentation [47]. Atypical presentations of GDLE have been reported in patients of differing ethnic groups; the morphological appearance of lesions in these patients varies from hyperpigmented macules to hyperkeratotic, hyperpigmented plaques with an erythematous border [53].

**Fig. 9:**
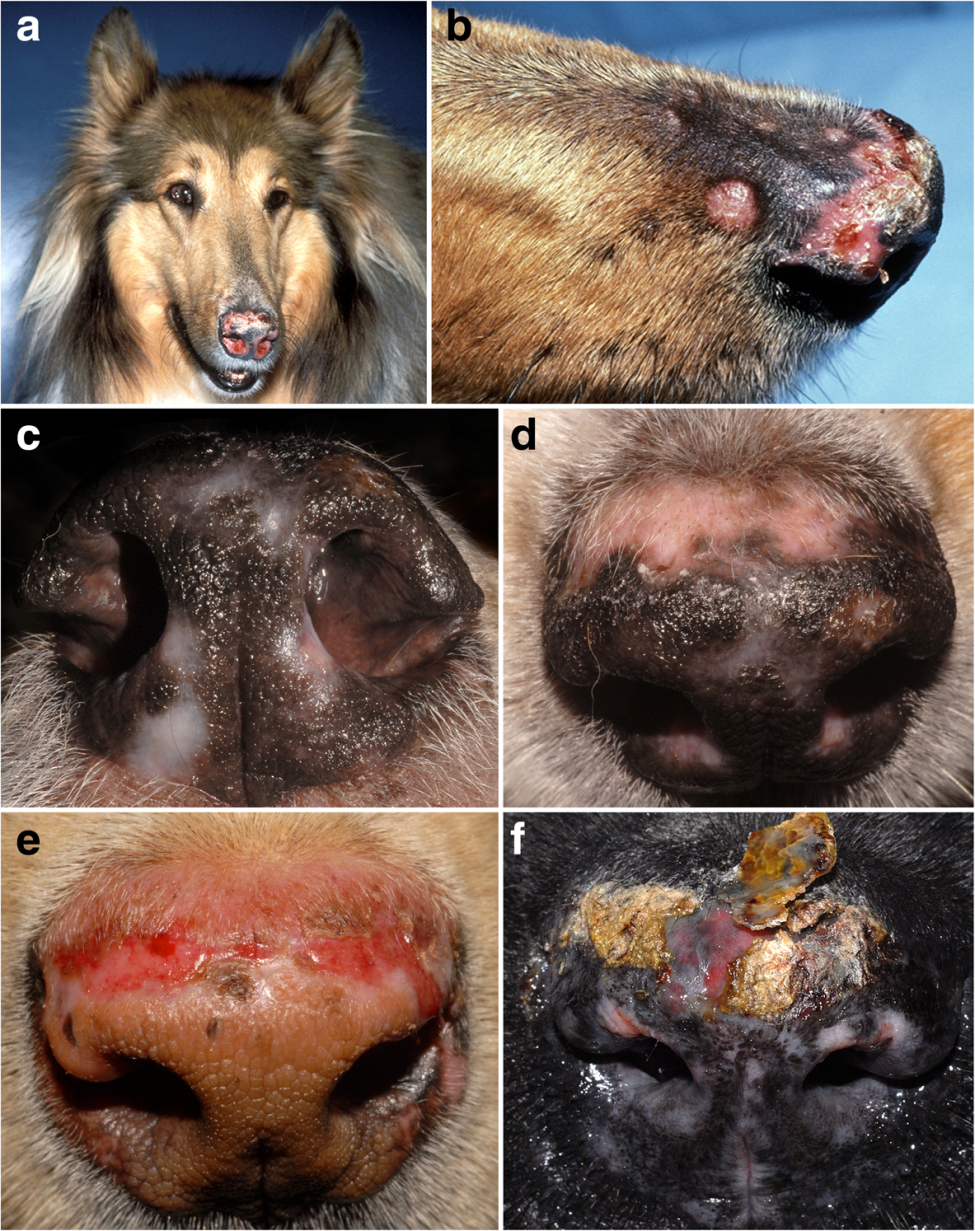
clinical characteristics of canine facial discoid lupus erythematosus. **a**, **b**: erythematous, depigmented, ulcerated, crusted and scarred nasal lesions of FDLE in a rough collie; a discoid lesion is visible in the proximal dorsal muzzle; (**c**, **d**): during the chronic phase of FDLE, depigmentation and scarring without inflammation are present; (**e**): erosions leading to scars in a Labrador with active FDLE; (**f**) depigmentation, scarring and crusting in a dog with FDLE. The presence of prominent inflammation often heralds a secondary bacterial colonization, like in the so-called MCP (courtesy of Petra Bizikova, NC State University, Raleigh)

The early skin lesions in canine FDLE consist of erythema, depigmentation and scaling that progress into erosions and ulcerations with atrophy and loss of the architecture of the nasal planum (Fig. 9a-f); crusting may be present if the epithelial integrity is damaged [3, 4]. Skin lesions usually affect the nasal planum (Fig. 9a-f) and might even involve the nares (Fig. 9c,d,f); several dogs exhibit additional skin lesions on the dorso-proximal muzzle (Fig. 9a,b), lips, periorbital skin and pinnae [3, 4]. Squamous cell carcinoma was reported to develop from chronic DLE nasal lesions in dogs [54], as in humans [55]. Pruritus has been reported to be variable in dogs with FDLE [3, 4].

Clinicians should remember that cutaneous (epitheliotropic) T-cell lymphomas can have localized lesions that affect the nose and could mimic those of FDLE. Other differential diagnosis for depigmentation and inflammation on the nasal planum are MCP and the uveodermatological syndrome, which resembles the Vogt-Koyanagi-Harada syndrome of humans. One should keep in mind that the "so-called MCP" is a poorly described disease that, if it were to even exist as a primary disease, is likely to occur secondarily to other diseases such as FDLE, MMP and MCLE and other nasal-targeting auto-immune and immune-mediated diseases.

**Fig. 10:**
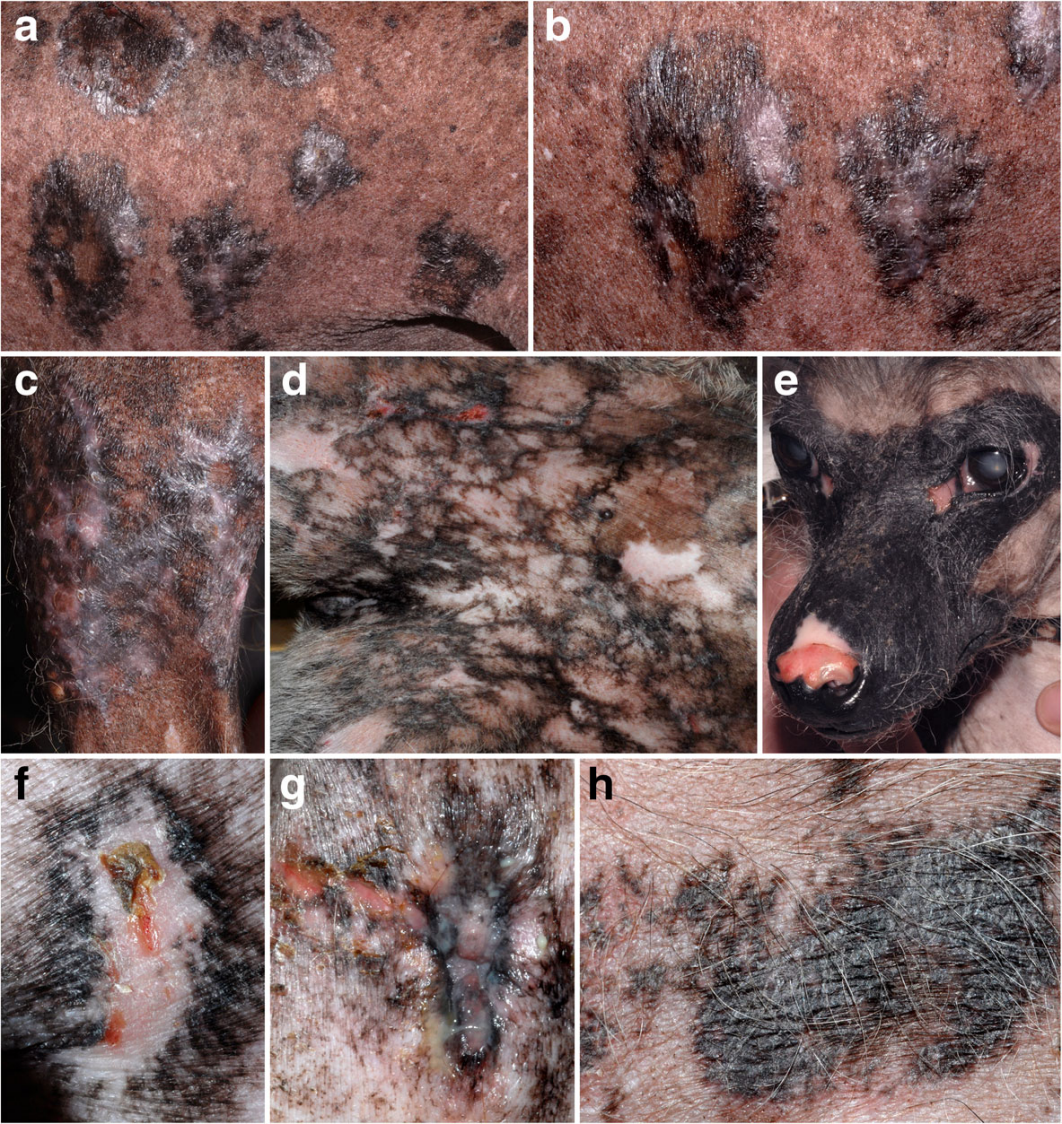
Clinical characteristics of canine generalized discoid lupus erythematosus. **a**, **b**: disc-shaped, annular and polycyclic plaques with hyperpigmentation, focal depigmentation and scarring on the thorax of a Chinese crested dog with GDLE; (**c**): large irregular plaque with dyspigmentation, scarring and erythema on the lateral knee of the same dog as in (**a**, **b**); **d**: reticulated depigmentation with occasional plaques and focal ulceration on the abdomen; (**e**): unusual “mask-like” bilateral and symmetric hyperpigmentation and dorsal proximal ulceration and scarring in another Chinese crested dog with GDLE; (**f**): same dog as in (**e**) – classic disc-shaped dyspigmented plaque with scarring and focal ulceration and crusting; (**g**): same dog as in (**e**) – anal and perianal dyspigmentation and scarring with focal ulceration; (**h**): large polycyclic hyperpigmented and scaly plaque on the abdomen of a crossbred dog with GDLE

Dogs with GDLE present with generalized or multifocal, annular (discoid) to polycyclic plaques with dyspigmentation, an erythematous margin, adherent scaling, follicular plugging and central alopecia; these predominate on the neck, dorsum and lateral thorax (Fig. 10a,f) [10]. In many of these dogs, the plaques evolved into ulcerations healing with a central atrophic or hypertrophic scar and dyspigmentation (depigmentation and hyperpigmentation) (Fig. 10a,f). Four of ten of the reported dogs (40%) had mucocutaneous regions involved with plaques usually appearing on or around the genitalia. An unusual pattern of reticulated (net-like) hyperpigmentation was visible on the ventral abdomen and lateral thorax in two of these cases, a feature also seen in other CCLE variants such as MCLE [9]. In the largest series of cases, systemic signs were not reported; pruritus and pain at the site of lesions were observed in four (40%) and three of ten dogs (30%), respectively [10]. There are only two canine skin diseases that could closely mimic GDLE: generalized (and often vaccine-induced) ischemic dermatopathies and the very rare hyperkeratotic EM (a.k.a. “old dog” EM).

##### Laboratory evaluation

In humans affected with the generalized variant of GDLE, a positive ANA titer is frequently found, and it represents a risk factor for development of SLE within five years after the initial diagnosis of skin lesions [56]. So far, out of the 104 dogs with classic FDLE included in the four largest series of cases, there were no reports of progression to SLE [3, 4, 45, 51]. Seven dogs with GDLE had a low positive ANA serum titer, but a progression with acquisition of additional criteria for SLE was not seen in any dog within the median follow up of 2.5 years (ranging 0.5 to 6 years) in the published series [10]. To our knowledge, the progression of a DLE variant to “clinical” SLE has been reported only in one dog [57].

##### Histopathology

The histology of DLE in dogs is similar to that of humans and is characterized by a lichenoid cell-rich, lymphocytic interface dermatitis reaction pattern with basal keratinocyte vacuolar degeneration, apoptosis, loss of basal cells and basement membrane thickening [1, 10].

**Fig. 11:**
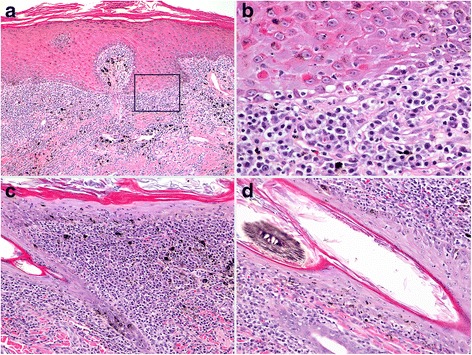
Histopathology of facial canine discoid lupus erythematosus. **a**: in a biopsy from the nasal planum, cell-rich, lymphocytic interface dermatitis is present with a prominent band-like (lichenoid) dermal infiltrate of lymphocytes and plasma cells. Pigmentary incontinence is moderate. 100× (**b**): inset box from image “a”, a short epidermal segment with well-developed interface change, where lymphocytes infiltrate predominantly the basal layer in conjunction with basal cell vacuolation, apoptosis, and loss. 400X (**c**): a similar interface reaction pattern affects the epidermis of haired skin in the dorsal nasal area. 200X (**d**): lymphocytic interface folliculitis and mural folliculitis of the hair follicle infundibulum. 200X

In canine FDLE, the interface reaction (vacuolar degeneration, apoptosis and loss of basal cells) is often subtle or mild in biopsy samples (Fig. 11a-c) [1, 10]. Only small areas might exhibit an active interface reaction and these lesions are easily missed, as nasal planum biopsies tend to be few and small. Interface changes can involve the follicular infundibula (Fig. 11d), when lesions extend off of the nasal planum; however, folliculitis has not been specifically investigated in canine FDLE. Pigmentary incontinence occurs secondarily to the interface reaction (Fig. 11a,b) but it is not specific to this type of injury and it can be found, persistent, in the nasal planum of dogs without concurrent nasal dermatitis [58, 59] Thickening of the basement membrane zone is patchy or multifocal but is not specific, as it occurs with other chronic inflammatory disorders of the nasal planum, such as leishmaniosis, where geographically relevant [60]. Superficial dermal fibrosis can be absent or range from mild-to-marked. Secondary bacterial colonization is common in FDLE and often complicates the histological diagnosis. These issues are compounded by the fact that, historically, the diagnosis of nasal-predominant “*canine DLE*” was given to dogs when microscopic examination of nasal planum skin biopsy specimens revealed a superficial dermal “*band-like*” pattern of inflammation rich in lymphocytes and plasma cells (a so-called “*lichenoid infiltrate*”), without any emphasis on the presence of an interface reaction. In fact, it is now believed that such “*lichenoid*” lymphocyte and plasma cell rich inflammation is a nonspecific inflammatory pattern seen in and near mucosae or related tissues (oral cavity, nasal planum, eyelids, genitalia, etc.). In a retrospective histological study of nasal dermatitis in dogs, a cell-rich lichenoid infiltrate was common, but only a small subset of subjects with nasal lesions exhibited the interface dermatitis associated with CLE [61].

**Fig. 12:**
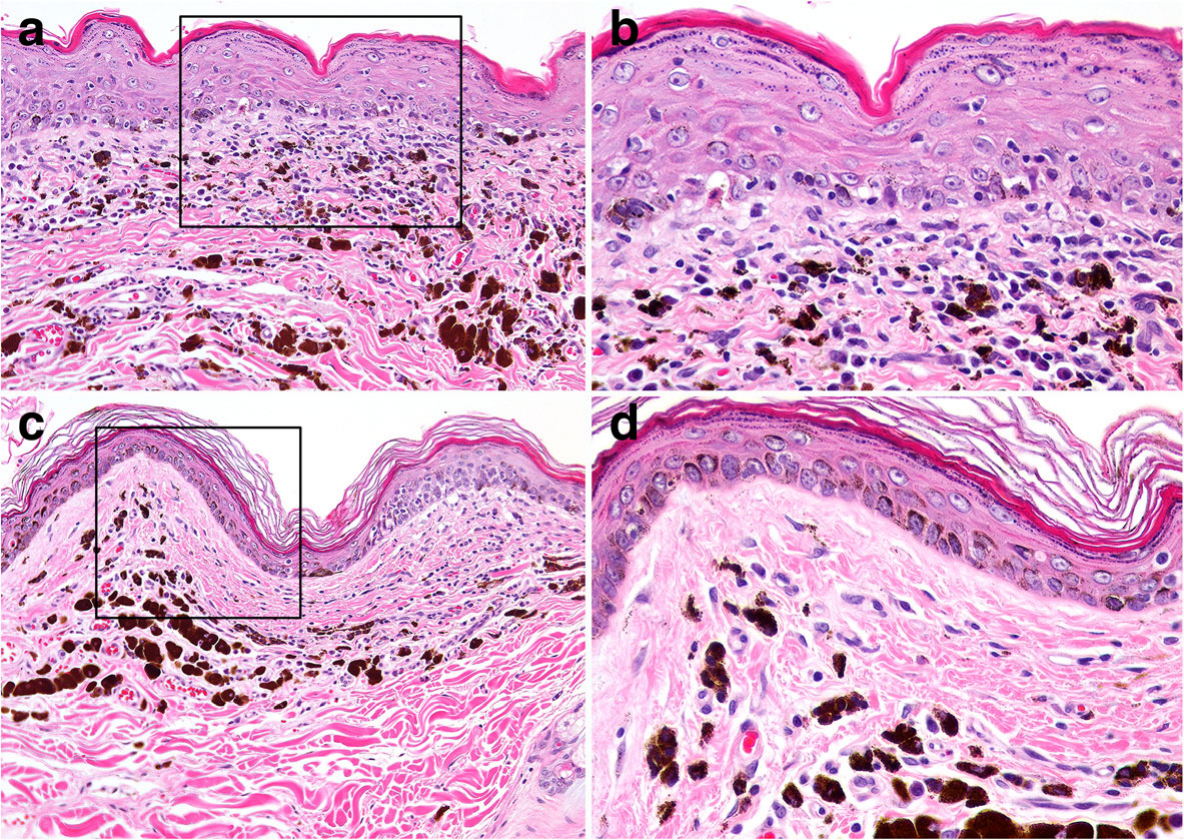
Histopathology of generalized canine discoid lupus erythematosus (**a**): in a skin biopsy from the trunk, a cell-rich lymphocytic interface dermatitis is present with prominent pigmentary incontinence. While epidermal atrophy (not shown) is classically seen in areas of prominent interface change, epidermal hyperplasia (shown here) can occur in chronic smoldering areas of lesions. 200X (**b**): inset box from image “a”, lymphocytes infiltrate predominately the basal layer in conjunction with basal cell vacuolation, apoptosis, and loss. 400X (**c**): some chronic lesions develop mild subepidermal fibrosis with a paucity of inflammation, while retaining pigmentary incontinence. 100X (**d**): inset box from image “c”, higher magnification image shows mild subepidermal fibrosis, few inflammatory cells and prominent pigmentary incontinence. 400X

In canine GDLE, in contrast to FDLE, the interface reaction is usually well developed, when an adequate number of biopsies are examined from the active margins of lesions (Fig. 12a,b) [10]. The epidermis may be atrophic or mildly hyperplastic (Fig. 12a,b) as a consequence of regional variation in severity of the interface reaction. Pigmentary incontinence can be pronounced, especially at the margins of lesions, where the interface reaction extends into zones of secondary hyperpigmentation induced by chronic inflammation (Fig. 12a-d). In chronic lesions, dermal fibrosis occasionally displaces the cell-rich inflammatory infiltrate from the superficial dermis (Fig. 12c,d). Cell-poor zones of lesion occasionally occur but often individual lymphocytes can be found in the basal layer of the epidermis in good numbers, with satellitosis of apoptotic basal keratinocytes. In GDLE, superficial epidermal apoptosis occurs, with or without lymphocytic satellitosis, which can erroneously suggest the diagnosis of erythema multiforme or morphologically related conditions. However, the collection of multiple biopsies reveals apoptosis to be most prominent in the basal epidermal layer in cases of GDLE.

In the recent case series of canine GDLE [10], alopecia occurred in nearly all patients; lymphocytic interface folliculitis involved the infundibulum and extended into the isthmus. A lymphocytic mural folliculitis was also common, but it was usually milder and involved the infundibulum, isthmus and inferior hair follicle segments, typically sparing the bulbs. This mural pattern mirrors that of human DLE, where it is also called a panfollicular pattern; it is usually minimally severe, but such a pattern is insufficiently described [62]. Sebaceous gland atrophy occurred in GDLE cases, where it was mostly mild and partial in biopsies but sometimes it was complete [10]. It should be noted that diagnostic biopsies typically focus on epidermal changes at the margins of skin lesions where hair follicle and sebaceous gland changes might not be fully developed.

##### Immunopathology

A linear deposition of IgG and IgM at the dermo-epidermal basement membrane zone (i.e. a positive LBT) of lesional skin was found in 90% of dogs with GDLE, and this proportion is similar to what is seen in human DLE lesions [10]. Interestingly, the most commonly detected immunoreactant deposited in one series of dogs with classic FDLE was C3 (90–100%), while IgG and IgM were revealed in 40–70% of cases, respectively [4]. In contrast, in the second case series, a positive LBT showed immunoglobulins (all classes together) and activated complement (C3) in 85–90% of 22 cases [3]. These variable results between canine localized and generalized DLE could be related to differences in tissue fixation techniques (frozen vs. formalin), antigen retrieval methods and/or immunofluorescence staining protocols that were performed 30 years apart. To investigate the value of performing DIF in canine CLE diagnostic work-up, further studies regarding the sensitivity and specificity of a positive LBT for the diagnosis of CLE variants are warranted.

##### Treatment and outcome

Besides the obvious need for photoprotection (sun avoidance), the 2017 update of the Cochrane systematic review of interventions for human DLE reported evidence for the benefit of a potent topical glucocorticoid and the oral drugs hydroxychloroquine and acitretin (a retinoid) [63] Furthermore, there was insufficient evidence for the efficacy of other interventions, such as topical calcineurin inhibitors (e.g. tacrolimus), [63].

Since 1992, antibiotics of the tetracycline family, with or without concurrent niacinamide (a.k.a. nicotinamide), have been suggested to be helpful for the treatment of canine immune-mediated skin diseases including FDLE. An initial report by White and colleagues showed that 14/20 (70%) dogs with FDLE had a good-to-excellent response using a tetracycline-niacinamide combination [64]; a recent retrospective study revealed a similar positive response rate in dogs with FDLE [45]. While tetracycline-niacinamide therapy is considered to be safe, tetracycline is no longer available commercially in many countries. Although tetracycline and doxycycline were shown to be relatively similar in their effectiveness to treat the so-called canine lupoid onychodystrophy, a poorly-understood onychitis [65], therapeutic equipotency data for other canine auto-immune and immune-mediated diseases, such as DLE are unavailable; additional studies are necessary to confirm the effectiveness of substituting doxycycline or minocycline for the tetracycline used beforehand to treat dogs with CLE.

Topical tacrolimus ointment has been used successfully for the topical treatment of canine FDLE. At first, Griffies and colleagues evaluated the use of 0.1% tacrolimus ointment applied topically to the lesional (facial) skin of ten dogs with DLE, most of these dogs receiving topical tacrolimus as an adjunctive therapy to oral glucocorticoids [66]. There was a positive response in eight dogs (80%), three of them having had an excellent improvement in skin lesions [66]. Recently, Messinger and colleagues conducted a randomized, double-blinded, placebo-controlled crossover study to evaluate the efficacy of a lower concentration of tacrolimus ointment (0.03%) in 19 dogs with FDLE [51]. Tacrolimus ointment, applied twice daily as monotherapy for up to 10 weeks, appeared safe and effective. A noticeable clinical improvement was seen in 13/18 (72%) of the dogs, whereas only three dogs receiving the placebo had lesions that improved. To summarize, limited outcome data suggest that topical tacrolimus ointment and/or a niacinamide-cyclin combination therapy should be considered as potentially effective therapeutic options for canine FDLE.

Skin lesions of canine GDLE appear to respond to a wide range of treatments but half of the patients experienced relapses upon the tapering of drug dosages. In a recent report [10], a remarkable improvement or a complete remission in GDLE skin lesions followed treatment with oral ciclosporin (mean 4.8 mg/kg once daily) along with a short course of glucocorticoids at treatment onset. Furthermore, oral hydroxychloroquine, in conjunction with topical 0.1% tacrolimus ointment application, helped induce and maintain remission of skin lesions in two dogs with GDLE [10].

### Lupus nonspecific skin diseases

In the Gilliam-Sontheimer CLE classification, lupus-nonspecific skin diseases are those that are not only present in the context of SLE, but also in other diseases; they do not have histopathology typical of CLE, however [11].

#### Cutaneous lesions associated with systemic lupus erythematosus

There is only scant information of skin lesions that occur during canine SLE. In the largest compilations of dogs with SLE, skin lesions were described in 33% [5] to 60% [67] of dogs, while oral ulcers were reported in 4 to 11% of cases, respectively [5, 67] Of note is that the first paper regrouped data from all cases published beforehand [5], while, in the other [67], skin lesions were not described in detail. In the first paper [5], Scott also reported characteristics on 26 new cases. In these cases, scaling (86% of the 14 dogs with dermatitis), mucocutaneous ulcerations (50%) and footpad ulcers and/or hyperkeratosis (42%) were most commonly seen [5]; two of 14 dogs (14%) exhibited lesions reportedly consistent with “lupus panniculitis” [5].

The microscopic lesions reported in 18 of these new cases were most commonly an interface dermatitis with variable inflammation [5]. While vasculitis was reported in only one case, the images of cell-poor interface dermatitis might represent sequelae of a lupus-associated vasculitis, a lupus-nonspecific skin disease; a lymphocytic septal panniculitis was observed in two dogs.

There is a clear need for more detailed descriptions of skin lesions associated with canine SLE. Future reports should also attempt to classify these lesions in the context of the human and canine CLE subsets described above.

#### Bullous systemic lupus erythematosus

In 1999, we reported a case that clinically resembled type I bullous SLE of humans (BSLE-I) [6]. In this four-year-old male castrated bichon frisé, erosions and crusts were present on the right elbow, axilla, thorax, pinna and labial commissures, and ulcers were also discovered on the footpad. Skin biopsies revealed subepidermal vesiculation and immunological testing uncovered skin-fixed and circulating IgG auto-antibodies that targeted type VII collagen in the epidermal basement membrane. As this dog also exhibited an intermittent fever, oral ulcers, a persistent proteinuria, a Coombs’ positive hemolytic anemia, a thrombocytopenia, a suspected pleuritis and hepatitis and elevated serum anti-nuclear autoantibodies, he was diagnosed has having concurrent SLE. The development of skin lesions associated with collagen VII auto-antibodies is normally typical of the disease epidermolysis bullosa acquisita, but, in the context of SLE, the diagnosis should change to type I bullous SLE [68]; as such, BSLE-I is a lupus-nonspecific skin disease.

## Conclusions

The number of canine CLE variants has increased since the princeps description of FDLE in dogs, nearly 40 years ago [1]. The accumulation of reports has led to the identification of predisposed breeds in many subsets and to a genetic linkage in the case of ECLE [35]. The recognition of additional subtypes of CCLE has revealed the overlap in some common skin lesions, which resemble those of human DLE, (i.e. polymorphic plaques with dyspigmentation, scarring and scaling). The new frontiers of canine CLE investigations will be to characterize and report atypical and crossover CLE variants—which are anecdotally mentioned being seen by colleagues—so that to add to the expanding phenotypic spectrum of canine CLE. Clinician-scientists are also urged to begin delving into the pathogenesis of CLE in dogs, to elucidate the genetic predisposition of the breed-specific variants (e,g. VCLE in collie breeds), the flare factors and the mechanisms of lesion formation. Finally, the usefulness of oral antimalarials for treatment of canine CLE variants should be investigated further.

## Abbreviations

ACLE: acute cutaneous lupus erythematosus
BP: bullous pemphigoid
BSLE-I: type-I bullous systemic lupus erythematosus
CCLE: chronic cutaneous lupus erythematosus
CLE: cutaneous lupus erythematosus
DLE: discoid lupus erythematosus
EBA: epidermolysis bullosa acquisita
ECLE: exfoliative cutaneous lupus erythematosus
EM: erythema multiforme
FDLE: facial discoid lupus erythematosus
GDLE: generalized discoid lupus erythematosus
GSHP: German shorthaired pointer
LBT: lupus band test
MCLE: mucocutaneous lupus erythematosus
MMP: mucous membrane pemphigoid
SCLE: subacute cutaneous lupus erythematosus
SLE: systemic lupus erythematosus
VCLE: vesicular cutaneous lupus erythematosus
